# Coexisting Graves' Disease and Papillary Thyroid Carcinoma: The Importance of Nodule Surveillance in Autoimmune Hyperthyroidism

**DOI:** 10.7759/cureus.103099

**Published:** 2026-02-06

**Authors:** Eliany Leon Figueredo, Ana B Cuni Hernandez, Annia Vega Fuentes, Juan T Vargas Rivera, Eduardo Oropesa, Ricardo Silvera

**Affiliations:** 1 Internal Medicine, Englewood Health Physician Network, Englewood, USA; 2 Family Medicine, IMG Academy, Bradenton, USA; 3 Family Medicine, Physician Network, Round Lake Beach, USA; 4 Family Medicine, Facultad de Ciencias Médicas de Pinar del Rio, Pinar del Rio, CUB; 5 Dermatology, Advanced Dermatology and Cosmetic Surgery, Lehigh Acres, USA; 6 Surgical Oncology, Baptist Health South Florida, Miami, USA

**Keywords:** braf mutation, graves' disease, papillary thyroid carcinoma, thyroid nodules, ultrasound surveillance

## Abstract

Graves' disease (GD) is an autoimmune disorder characterized by hyperthyroidism and is commonly associated with diffuse thyroid enlargement. The coexistence of thyroid nodules in GD presents diagnostic and management challenges, particularly when malignancy is a concern. We report a 44-year-old woman with asymptomatic biochemical hyperthyroidism detected during routine screening. Initial ultrasound revealed a subcentimeter right thyroid nodule (TI-RADS TR4), for which surveillance was recommended. Follow-up imaging demonstrated interval growth, prompting fine-needle aspiration that was suspicious for papillary thyroid carcinoma (PTC) (Bethesda V). Molecular analysis revealed a rare BRAF mutation with an estimated ~70% probability of malignancy. The patient underwent total thyroidectomy with central neck dissection, confirming a 1.1 cm classic PTC with microscopic nodal metastasis (pT1bN1a). She recovered uneventfully and remains under endocrinology follow-up. This case underscores the importance of structured ultrasound surveillance in patients with GD, even in the absence of clinical symptoms. A multimodal diagnostic approach incorporating imaging, cytology, and molecular analysis can facilitate early detection and informed risk stratification, supporting timely and appropriate management.

## Introduction

Graves' disease (GD) is the most common organ-specific autoimmune thyroid disorder and the leading cause of hyperthyroidism worldwide. It accounts for approximately 50-80% of hyperthyroid cases and affects nearly 0.5% of the US population, with a marked female predominance (about 2% of women versus 0.2% of men). Most patients present between the third and sixth decades of life, and recent epidemiological data estimate an annual incidence of roughly 20 per 100,000 individuals in Western populations, underscoring the substantial healthcare burden of this condition. GD is characterized by the presence of thyrotropin (TSH) receptor-stimulating antibodies, which drive diffuse thyroid hyperplasia, excessive thyroid hormone production, and frequently goiter formation. Beyond inducing hyperthyroidism, these immunoglobulins may act as growth factors for thyrocytes, raising questions about their potential role in the development of thyroid nodules and neoplasms [1].

From a diagnostic and management standpoint, the coexistence of thyroid nodules in GD presents a significant clinical challenge. GD typically manifests as a diffusely enlarged and hypervascular gland, the so-called "thyroid inferno", which can obscure discrete nodules on physical examination. Ultrasound-based studies have demonstrated that a substantial proportion of GD patients harbor thyroid nodules that are not clinically apparent. Consequently, current guidelines and expert recommendations emphasize the importance of high-resolution ultrasonography and risk-stratified fine-needle aspiration (FNA) biopsy based on nodule size and suspicious sonographic features [2].

As familiarity with thyroid nodule classification systems varies across specialties, outlining the diagnostic framework offers important context for understanding this case. The American College of Radiology Thyroid Imaging Reporting and Data System (ACR TI-RADS) is a standardized scoring method that estimates malignancy risk based on nodule characteristics, including composition, echogenicity, margins, shape, and the presence of calcifications, ultimately categorizing nodules from TR1 (benign) to TR5 (highly suspicious). Similarly, the Bethesda System for Reporting Thyroid Cytopathology provides a uniform diagnostic structure for FNA specimens, ranging from Category I (non-diagnostic) to Category VI (malignant) [3].

Historically, a hyperthyroid state was thought to be "protective" against thyroid cancer, creating a false sense of security in clinical practice. Older studies reported very low malignancy rates in hyperfunctioning nodules, reinforcing the notion that toxic nodules were rarely cancerous. However, contemporary research contradicts this belief. Papillary thyroid carcinoma (PTC), the most common type of thyroid malignancy accounting for approximately 80% of all thyroid cancers, generally exhibits indolent growth but has a propensity for regional lymph node metastasis. Recent studies indicate that, in patients with GD, the presence of thyroid nodules increases the risk of PTC, highlighting the importance of recognizing this association for the timely diagnosis and appropriate management of nodular GD [4].

Clinicians must carefully balance the timely treatment of hyperthyroidism with appropriate cancer surveillance, a task complicated by overlapping clinical and imaging features. Failure to identify a malignant nodule in the context of GD may delay definitive surgical management in patients who might otherwise be treated with antithyroid medications or radioactive iodine alone. Conversely, increased imaging surveillance raises concerns about overdiagnosis of small, indolent tumors, highlighting the need for nuanced clinical judgment. This diagnostic and therapeutic conundrum makes each confirmed case of coexisting GD and PTC particularly instructive [5].

Here, we present the case of a 44-year-old woman with GD and concomitant PTC, highlighting the clinical relevance of this association. This case emphasizes that thyroid nodules in GD warrant careful evaluation despite a hyperfunctioning gland and underscores the need for integrated management that addresses both hyperthyroidism and structural thyroid abnormalities.

## Case presentation

A 44-year-old woman was referred to the endocrinology clinic after routine laboratory testing during a preventive health examination which revealed biochemical hyperthyroidism. She had no prior history of thyroid disease. At presentation, she denied weight loss, palpitations, tremor, heat intolerance, anxiety, sleep disturbance, increased bowel movements, neck pain, or compressive symptoms. There were no history of head or neck radiation exposure and no family history of thyroid disease or thyroid malignancy.

Initial laboratory testing revealed biochemical evidence of hyperthyroidism, with thyroid autoantibodies suggestive of underlying autoimmune thyroid disease. The patient remained clinically asymptomatic, and physical examination, including thyroid palpation, was unremarkable, without goiter or cervical lymphadenopathy. Laboratory results are summarized in Table 1.

**Table 1:** Initial laboratory findings TSH: thyroid-stimulating hormone; fT4: free thyroxine; T3: total triiodothyronine; TPOAb: thyroid peroxidase antibodies; TgAb: thyroglobulin antibodies; µIU/mL: micro-international units per milliliter; ng/dL: nanograms per deciliter; ng/mL: nanograms per milliliter; IU/mL: international units per milliliter

Test	Result	Reference range	Interpretation
TSH	<0.005 µIU/mL	0.4-4.0 µIU/mL	Markedly suppressed, consistent with hyperthyroidism
Free T4 (fT4)	2.4 ng/dL	0.8-1.8 ng/dL	Elevated, confirming biochemical hyperthyroidism
Total T3 (T3)	2.52 ng/mL	0.6-1.8 ng/mL	Elevated, consistent with hyperthyroidism
TPOAb	4.7 IU/mL	<5.6 IU/mL	Within normal limits
TgAb	4.8 IU/mL	<4.0 IU/mL	Mildly elevated, indicative of thyroid autoimmune activity

**Figure 1:**
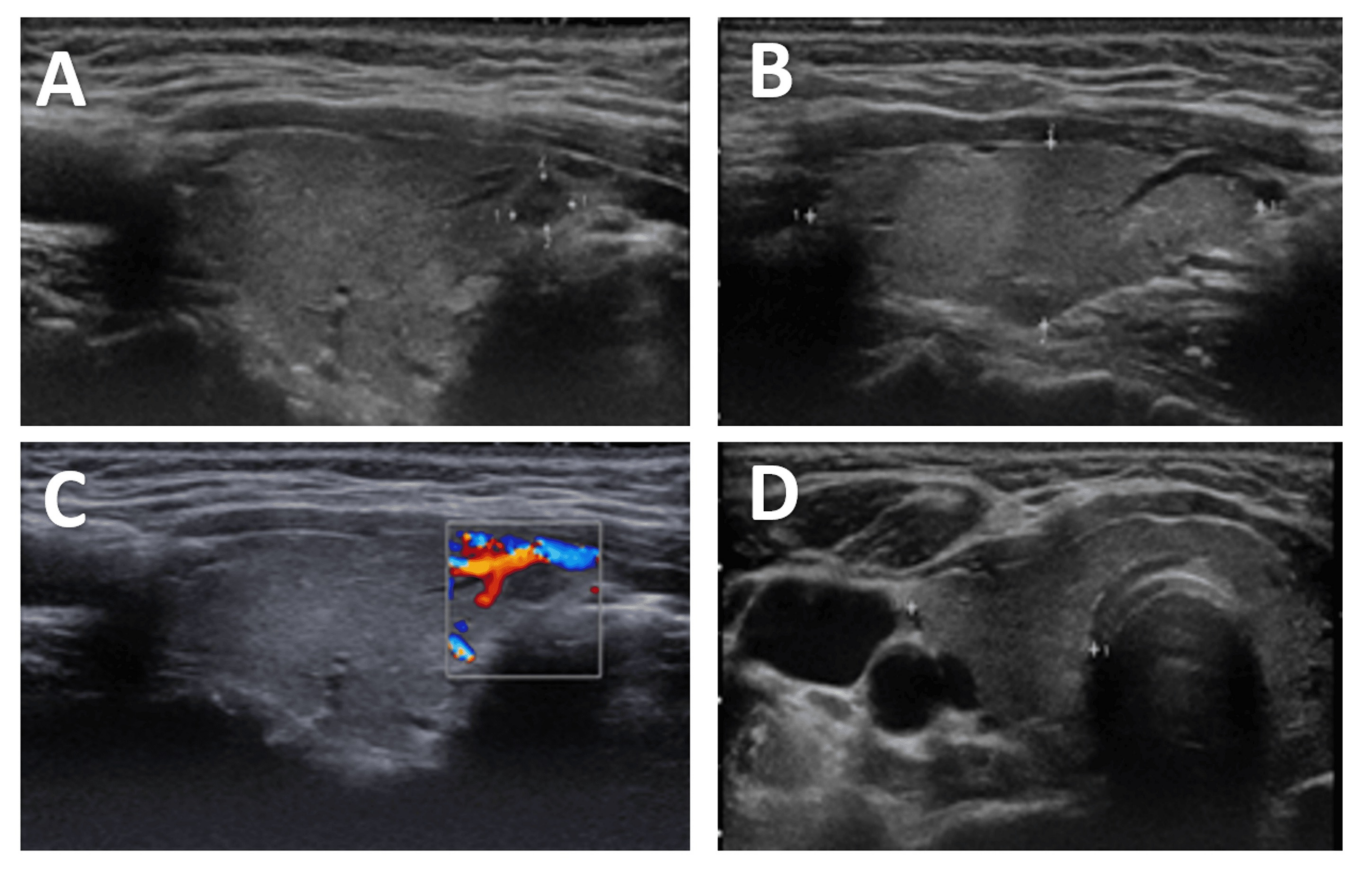
Thyroid ultrasound findings at the initial evaluation (A) Transverse grayscale ultrasound image of the right thyroid lobe demonstrating a normal-sized gland with homogeneous echotexture and a solitary mildly hypoechoic solid nodule in the anteromedial aspect of the right lower pole. (B) Longitudinal grayscale view of the same nodule showing smooth margins without microcalcifications or evidence of extrathyroidal extension. (C) Color Doppler ultrasound demonstrating mild internal and peripheral vascularity within the nodule. (D) Transverse ultrasound image showing the nodule in relation to surrounding cervical structures, with no abnormal cervical lymph nodes identified.

A thyroid ultrasound demonstrated a normal-sized gland with homogeneous echotexture. A solitary mildly hypoechoic solid nodule measuring 0.5×0.4×0.4 cm was identified in the anteromedial aspect of the right lower pole (Figure 1). The nodule had smooth margins, no microcalcifications, and no evidence of extrathyroidal extension, corresponding to ACR TI-RADS Category TR4. No abnormal cervical lymph nodes were visualized. Given the small size of the nodule and absence of high-risk sonographic features, FNA was not indicated at that time, and surveillance imaging was recommended.

**Figure 2:**
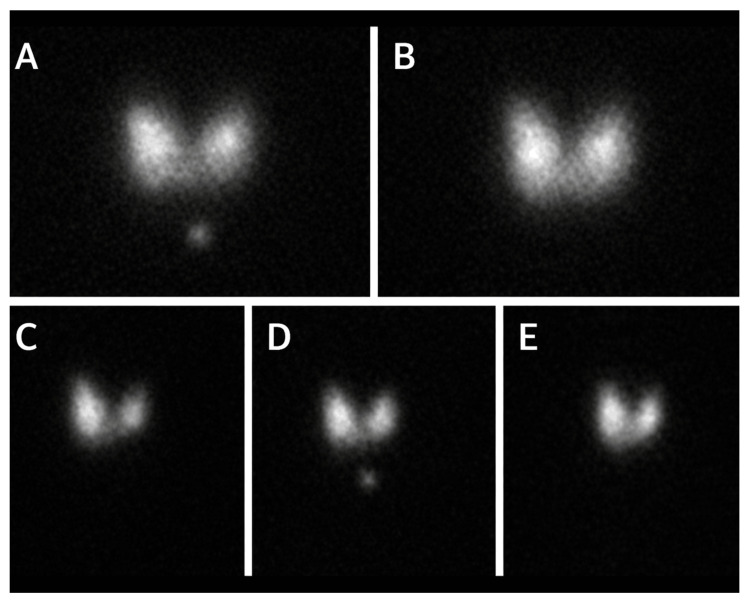
Radioactive iodine uptake and scan demonstrating diffusely increased tracer activity consistent with Graves' disease (A) Anterior thyroid scintigraphy image showing homogeneous radiotracer distribution throughout both lobes. (B) Additional anterior projection confirming uniform tracer uptake without focal hyperfunctioning or nonfunctioning nodules. (C) Right anterior oblique view demonstrating symmetric uptake within the right lobe. (D) Left anterior oblique view showing comparable uniform activity in the left lobe. (E) Supplemental projection confirming absence of focal defects or asymmetric radiotracer accumulation.

To further evaluate the etiology of her hyperthyroidism, a radioactive iodine uptake and scan was obtained. The study demonstrated homogeneous tracer uptake throughout both thyroid lobes, without focal hyperfunctioning or nonfunctioning nodules. Radioactive iodine uptake was markedly elevated, measuring 65.3% at six hours (reference range 4-15%) and 87.8% at 24 hours (reference range 15-30%), findings consistent with GD (Figure 2). 

Given her lack of symptoms, beta-adrenergic blockade was deferred. Treatment options, including antithyroid medications, radioactive iodine ablation, and surgery, were discussed. The patient was initially managed conservatively and later started on methimazole. Follow-up laboratory studies three months later showed near normalization of thyroid function tests, with a TSH of 0.043 µIU/mL, free T4 of 0.7 ng/dL, and total T3 of 0.76 ng/mL. Liver function tests were within normal limits. The patient continued to feel well and remained asymptomatic.

**Figure 3:**
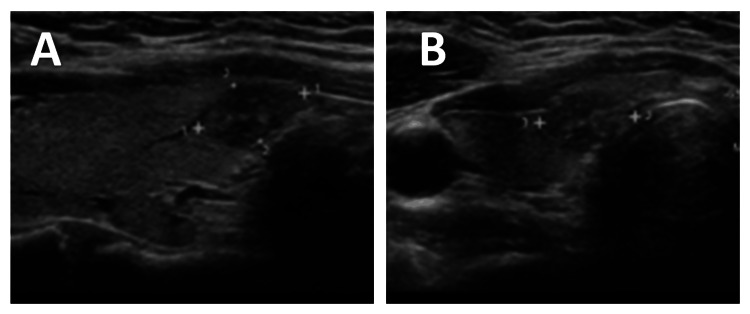
Surveillance thyroid ultrasound demonstrating interval nodule growth (A) Transverse grayscale ultrasound image of the right thyroid lower pole showing interval enlargement of the previously identified nodule with a mildly heterogeneous and predominantly hypoechoic appearance. (B) Longitudinal grayscale view of the same nodule confirming interval growth compared with prior imaging.

Surveillance thyroid ultrasonography performed one year later demonstrated interval growth of the previously identified right lower pole nodule, now measuring 1.1×0.6×1 cm, with a mildly heterogeneous and predominantly hypoechoic appearance (Figure 3). Because the nodule increased from 0.5 cm to 1.1 cm over one year, a meaningful interval change for a TI-RADS TR4 lesion, ultrasound-guided FNA was recommended according to established risk-stratified criteria.

**Figure 4:**
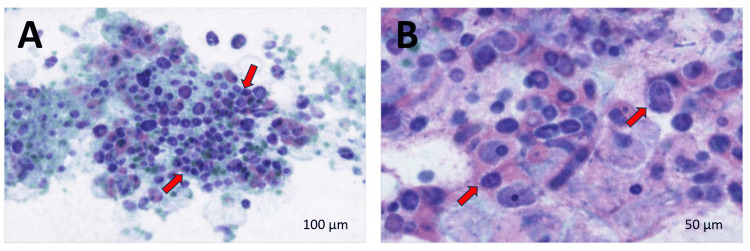
Diff-Quik-stained fine-needle aspiration cytology of the thyroid nodule (A) Low-power view showing atypical follicular cells arranged in three-dimensional clusters and syncytial sheets with thick colloid in the background (red arrows). (B) Higher-power view highlighting enlarged oval nuclei with nuclear grooves and rare intranuclear pseudoinclusions (red arrows). Scale bars: A=100 µm; B=50 µm

Cytologic evaluation of the aspirate was suspicious for PTC (Bethesda System Category V). Smears demonstrated atypical follicular cells arranged in three-dimensional clusters and syncytial sheets, with enlarged oval nuclei, nuclear grooves, and rare intranuclear pseudoinclusions, with thick colloid in the background (Figure 4). Molecular analysis using ThyroSeq revealed a rare BRAF mutation, which has been reported to confer an approximately 70% probability of malignancy and is more commonly associated with follicular or solid variants of PTC. 

Given the cytologic findings, molecular results, and underlying GD, the patient was referred to endocrine surgery. After multidisciplinary discussion, she underwent total thyroidectomy with central neck (level VI) lymph node dissection. The procedure was uncomplicated.

**Figure 5:**
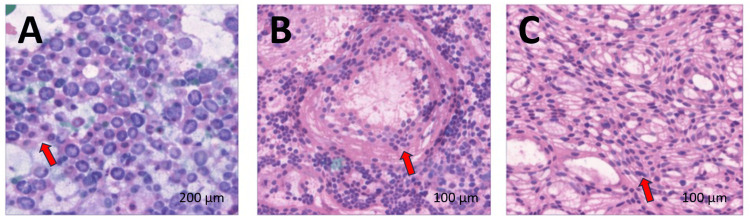
Hematoxylin and eosin-stained sections of papillary thyroid carcinoma and background thyroid tissue (A) Low-power view demonstrating classic papillary thyroid carcinoma architecture with fibrovascular cores and characteristic nuclear features (red arrow). (B) Higher-power view showing a microscopic metastatic focus of papillary thyroid carcinoma within a central compartment lymph node (red arrow). (C) Background thyroid tissue with chronic lymphocytic thyroiditis (red arrow). Scale bars: A=200 µm; B=100 µm; C=100 µm

Final surgical pathology confirmed classic PTC measuring 1.1 cm in greatest dimension, with negative surgical margins and no lymphovascular invasion. One of the five central compartment lymph nodes demonstrated a microscopic metastatic focus measuring 0.5 mm, without extranodal extension. Background thyroid parenchyma showed features of chronic thyroiditis. The tumor was staged as pT1bN1a according to the American Joint Committee on Cancer (AJCC) eighth edition staging system (Figure 5).

Postoperatively, the patient recovered without complications and denied symptoms of hypocalcemia. She was started on levothyroxine 88 µg daily. At follow-up, thyroid function tests demonstrated biochemical euthyroidism. Surveillance laboratory testing showed undetectable thyroglobulin antibodies and low serum thyroglobulin levels (0.5 ng/mL). She continues regular follow-up with endocrinology and endocrine surgery, with consideration of radioactive iodine ablation based on ongoing risk stratification. 

## Discussion

GD is an autoimmune disorder characterized by complex humoral and cellular immune responses directed against multiple thyroid antigens, most notably the thyroid-stimulating hormone receptor (TSHR). Circulating IgG autoantibodies against TSHR lead to the persistent activation of cyclic AMP-mediated signaling pathways in thyroid follicular cells, resulting in increased thyroid hormone synthesis, iodine uptake, cellular proliferation, and glandular hypertrophy, with the subsequent suppression of pituitary TSH secretion. Although antibodies against thyroglobulin, thyroid peroxidase, and the sodium-iodide symporter are frequently detected, they primarily function as markers of autoimmune thyroid disease rather than direct mediators of hyperthyroidism. Histologically, intrathyroidal lymphocytic infiltration is an early and characteristic feature of GD and reflects an inflammatory microenvironment in which thyroid epithelial cells actively participate through cytokine production, the expression of adhesion molecules, and immune regulatory pathways. Chronic immune stimulation, increased follicular cell turnover, and cytokine-mediated inflammation have been proposed as factors that may contribute to a thyroid microenvironment associated with, but not necessarily causative of, malignant transformation. However, a direct causal relationship between GD and thyroid carcinoma has not been definitively established [6].

GD is the most common cause of hyperthyroidism and is typically managed with antithyroid medications, radioactive iodine ablation, or surgical thyroidectomy, with treatment selection individualized based on patient characteristics, disease severity, and coexisting thyroid pathology. One clinically relevant consideration is the presence of thyroid nodules, as an increasing body of evidence suggests that nodular GD represents a subgroup with a higher risk of thyroid malignancy [7]. 

Thyroid nodules are common in the general population, with a prevalence exceeding 50%, though only a small proportion are malignant. In patients with GD, however, several contemporary studies have demonstrated that the coexistence of thyroid nodules significantly modifies cancer risk [8]. Multicenter cohorts of surgically treated patients with GD report a prevalence of PTC exceeding 35%, increasing to nearly 50% among patients with synchronous nodular disease, while remaining substantially lower in patients with diffuse, non-nodular GD; however, these estimates largely derive from surgical cohorts and may overestimate the true prevalence in the general GD population due to selection bias. Meta-analyses and umbrella reviews further support an approximately fivefold increased risk of thyroid cancer when nodules are present, regardless of whether they are solitary or multiple. However, reported risk estimates vary across studies due to differences in cohort composition, the inclusion of surgical versus non-surgical populations, diagnostic thresholds, and follow-up duration. These findings underscore that, although GD is classically regarded as a diffuse thyroid disorder, the presence of nodules is a critical modifier of malignancy risk and warrants careful evaluation and longitudinal surveillance [9]. In the present case, the identification of thyroid nodules in a patient with GD highlights the importance of careful evaluation and appropriate diagnostic workup, as coexisting malignancy, although uncommon, may significantly influence therapeutic decision-making.

The clinical presentation is highly variable. While many patients experience symptoms related to thyroid hormone excess, such as palpitations, tremor, heat intolerance, and weight loss, others may demonstrate overt biochemical hyperthyroidism with minimal or absent clinical manifestations. This discordance likely reflects individual variability in hormone sensitivity and adaptive mechanisms and highlights the limitations of symptom-based assessment alone [10]. Our patient remained entirely asymptomatic despite persistently suppressed TSH and elevated free T4 and total T3 on serial testing, consistent with biochemically overt but clinically silent hyperthyroidism. Functional evaluation with radioactive iodine uptake and scan demonstrated markedly elevated, homogeneous uptake, supporting the diagnosis of GD and helping exclude alternative etiologies such as thyroiditis or toxic nodular disease. This diagnostic approach aligns with current guidelines recommending functional imaging or TSHR antibody testing when the clinical picture is atypical or inconclusive.

Thyroid ultrasound plays a central role in the evaluation, even in the absence of palpable goiter or compressive symptoms, as it allows the detection and risk stratification of thyroid nodules using standardized systems such as the ACR TI-RADS [11]. Ultrasound surveillance of thyroid nodules is a cornerstone in the early identification of structural thyroid abnormalities that may warrant further evaluation. Given the high prevalence of benign nodules and the often indolent course of differentiated thyroid carcinoma, longitudinal ultrasound follow-up enables the detection of interval growth or evolving morphologic features associated with malignancy, such as increasing hypoechogenicity, irregular margins, microcalcifications, or suspicious cervical lymphadenopathy, while minimizing unnecessary invasive procedures. Importantly, ultrasound surveillance remains essential even after an initially benign or low-risk assessment, as interval changes may prompt repeat biopsy and allow the timely diagnosis of malignancy that was not evident on initial evaluation [12].

In this patient, the initial ultrasound identified a subcentimeter hypoechoic solid nodule classified as TI-RADS TR4, for which surveillance rather than immediate FNA was appropriate according to established guidelines. Interval growth detected on follow-up ultrasound prompted FNA, which yielded cytology suspicious for PTC (Bethesda System Category V). According to the 2023 Bethesda System for Reporting Thyroid Cytopathology, Category V represents a high-risk diagnostic group with an estimated malignancy risk of approximately 60-75% in the absence of molecular testing. This category is intended to balance diagnostic accuracy by minimizing false-positive malignant diagnoses while identifying nodules that warrant prompt surgical management [3]. The updated Bethesda framework emphasizes the integration of cytologic findings with clinical, radiologic, and molecular data, a principle that proved particularly relevant in this case. This multidimensional approach, integrating biochemical, functional, and morphologic studies, is particularly valuable because it demonstrates that a classic biochemical profile of GD, even in asymptomatic patients, does not exclude coexisting malignancy. 

Molecular profiling has become an important adjunct in the evaluation of indeterminate or suspicious thyroid nodules. Activating mutations in the BRAF gene, most commonly BRAF V600E, drive papillary thyroid carcinogenesis through the constitutive activation of the MAPK/ERK signaling pathway, resulting in sustained cellular proliferation and impaired differentiation. Less common non-V600E BRAF mutations are increasingly recognized and are often associated with follicular-patterned or solid variants of PTC [13]. In the present case, molecular analysis revealed a rare BRAF mutation detected by ThyroSeq, conferring an estimated ~70% probability of malignancy, consistent with the cytologic findings suspicious for PTC. Recent clinical and molecular studies support the use of next-generation sequencing classifiers to refine malignancy risk and inform surgical decisions, particularly when cytology is indeterminate or suspicious. BRAF-mutated PTC have been associated with more aggressive clinicopathologic features, including extrathyroidal extension, lymph node metastasis, and increased risk of recurrence [14]. However, clinical behavior among BRAF-mutated tumors is heterogeneous, and not all exhibit aggressive features; outcomes depend on additional molecular, histologic, and patient-specific factors. In this patient, final pathology demonstrated microscopic central lymph node metastasis (pN1a) despite a primary tumor measuring 1.1 cm, underscoring that certain BRAF mutations may predispose to early regional spread even in small tumors.

Sustained MAPK pathway activation has also been linked to the reduced expression of thyroid-specific genes involved in iodine metabolism, such as the sodium-iodide symporter, potentially contributing to radioiodine refractoriness in a subset of patients. These molecular characteristics carry therapeutic relevance, as targeted therapies using BRAF and MEK inhibitors have demonstrated benefit in advanced or radioiodine-refractory thyroid carcinoma and may restore iodine avidity in selected cases. Thus, molecular profiling provides clinically actionable information that may influence both initial management and long-term therapeutic strategies [15]. This case highlights the clinical importance of BRAF mutation testing in PTC arising in the context of GD, even when conventional clinicopathologic features suggest low-risk disease. Integration of cytology, molecular profiling, and histopathologic findings enables more accurate risk stratification and supports a precision-based approach to thyroid cancer management.

Although the prevalence of PTC is increased in patients with GD and nodular disease, the prognostic implications of this association remain controversial. Earlier studies suggested a more aggressive tumor phenotype in GD-associated PTC; however, more recent analyses that adequately control for confounding factors challenge this assumption. Propensity score-matched studies demonstrate comparable recurrence-free survival between patients with PTC with and without GD when adjusted for tumor size and lymph node involvement, suggesting that GD itself is not an independent determinant of adverse prognosis. Rather, established clinicopathologic factors remain the primary predictors of outcome [16].

Many previously reported cases describe PTC diagnosed incidentally following thyroidectomy performed for refractory hyperthyroidism [17]. In contrast, the present case illustrates the value of longitudinal ultrasound surveillance in detecting clinically significant malignancy in an asymptomatic patient with GD and an initially low-risk subcentimeter nodule. The identification of microscopic nodal metastasis further emphasizes that a benign clinical presentation should not preclude vigilant follow-up. Overall, this case highlights the importance of systematic ultrasound surveillance in patients with GD, even when symptoms are absent, and demonstrates how the integration of sonographic follow-up, Bethesda V cytology, and molecular profiling enables timely diagnosis, appropriate surgical management, and refined risk stratification through a precision-based approach.

This report is limited by its nature as a single case, which restricts generalizability and precludes causal inference regarding the relationship between GD and PTC. There is also potential surveillance bias, as patients with GD who undergo more frequent ultrasound monitoring may have a higher likelihood of detecting small or subclinical cancers compared with less closely monitored populations. In addition, the absence of longitudinal immunologic data, such as serial TSHR antibody titers, limits the ability to assess potential correlations between immunologic activity and structural disease progression. Despite these limitations, the case offers valuable clinical insight by emphasizing the diagnostic importance of ultrasound surveillance in nodular GD and reinforcing awareness of this clinically relevant association.

## Conclusions

GD can coexist with PTC, even in asymptomatic patients with initially low-risk thyroid nodules. The presence of nodules in GD significantly increases the risk of malignancy, underscoring the importance of systematic evaluation, longitudinal ultrasound surveillance, and risk-stratified FNA to enable early detection and timely intervention.

Molecular profiling, including BRAF mutation analysis, provides valuable prognostic and therapeutic information, allowing for precision-based management. This case highlights the importance of integrating clinical, biochemical, imaging, cytologic, and molecular data to guide individualized care in patients with nodular GD and reinforces vigilance for coexisting malignancy.
